# Procyanidin B2 Attenuates Sepsis-Induced Acute Lung Injury via Regulating Hippo/Rho/PI3K/NF-κB Signaling Pathway

**DOI:** 10.3390/ijms24097930

**Published:** 2023-04-27

**Authors:** Go Oun Kim, Dong Ho Park, Jong-Sup Bae

**Affiliations:** 1 College of Pharmacy, Research Institute of Pharmaceutical Sciences, Kyungpook National University, Daegu 41566, Republic of Korea; rhdns9231@gmail.com; 2 Department of Ophthalmology, School of Medicine, Kyungpook National University, Kyungpook National University Hospital, Daegu 41944, Republic of Korea; dongho_park@knu.ac.kr

**Keywords:** procyanidin B2, sepsis, acute lung injury, hippo, rho

## Abstract

Acute lung injury (ALI) is a frequent and challenging aspect of sepsis that currently lacks effective treatments. Procyanidin B2 (PB2) has anti-inflammatory and antioxidant properties. The aim of this study was to determine the effectiveness and mechanism of action of PB2 in treating sepsis-induced ALI using animal experiments. A sepsis-induced ALI mouse model was used by administering lipopolysaccharide (LPS) and then evaluating the levels of inflammatory cytokines and lung injury through measurements of cytokine levels using enzyme-linked immunosorbent assay (ELISA), Western blot and real-time PCR, as well as by the examination of relevant signaling pathways. The animal experiments showed that PB2 protected the lungs from injury caused by LPS and reduced the levels of various inflammatory cytokines in both the serum and lung tissue. Western blot analysis showed that PB2 reduced the expression of TLR4/NF-κB and increased the expression of PI3K/Akt, and also inhibited the Hippo and Rho signaling pathways. The results of the study showed that PB2 helps to treat sepsis-induced ALI by controlling cytokine storms and reducing inflammation by altering the expressions of the TLR4/NF-κB, PI3K/Akt, Hippo and Rho signaling pathways. This research provides a foundation for the further investigation of PB2’s mechanism and its potential use in treating sepsis.

## 1. Introduction

Sepsis is a serious and life-threatening condition that occurs when the body overreacts to an infection [1]. The lung is particularly susceptible to injury during sepsis and is often the first organ to fail. This can lead to respiratory failure and the development of acute lung injury (ALI) and acute respiratory distress syndrome (ARDS), increasing the risk of multiorgan dysfunction [2,3]. The severity of sepsis is due to a cytokine storm, which is an excessive immune response to infections, autoimmune disorders, and other conditions [1,4]. This is characterized by elevated levels of inflammatory cytokines, such as interleukin (IL), interferon (IFN), chemokine, tumor necrosis factor (TNF), and colony-stimulating factor (CSF), but there are currently no effective drugs to treat sepsis-induced ALI [4]. The primary therapies consist of noninvasive and invasive breathing assistance, repositioning onto the stomach, muscle relaxation medication, external oxygenation through a membrane, medication to reduce inflammation, and care that provides comfort and assistance [5]. 

At present, there are no drugs or treatments that can cure ALI caused by sepsis. The primary therapies available include mechanical ventilation that can be either invasive or noninvasive, repositioning onto the stomach, medication to relax muscles, external oxygenation through a membrane, medication to reduce inflammation, and care that provides comfort and assistance [5]. Among these, anti-inflammatory therapy is an essential and effective treatment. The use of corticosteroids to treat sepsis-induced ALI remains controversial [6]. Although corticosteroids can improve lung function, reduce inflammation, and increase the survival rate of ALI, their clinical application often leads to various side effects, such as hyperglycemia, hypokalemia, dyslipidemia, osteoporosis, myopathy, and immunosuppression. 

The drug known as recombinant activated protein C, also referred to as Xigris by Eli Lilly, was granted approval by the Food and Drug Administration (FDA) and the European Medicine Agency (EMA) in 2001 and 2002, respectively, for treating severe sepsis and septic shock [7]. This drug possesses various properties including anticoagulant, anti-inflammatory, barrier-protective, and fibrinolytic properties; however, in October 2011, it was removed from the market due to its negative side effects and the lack of beneficial effects on mortality rates in trials such as PROWESS and septic shock (PROWESS-SHOCK) trials [8]. Bleeding is a frequent negative reaction linked to APC, resulting from the breakdown of the procoagulant substances Va and VIIIa [8]. This is in line with the antithrombotic nature of APC, and there are no approved drugs for severe sepsis as a result [9]. Hence, it is crucial to discover fresh and efficient targets for treatment to fight sepsis. 

Procyanidin B2 (PB2), a type of antioxidant belonging to the proanthocyanidin flavonoid polyphenol class, is found in various plants, such as grape seeds, berries, cocoa, and tea. and has multiple pharmacological properties, such as anti-oxidative and anti-inflammatory effects [10,11,12]. Previous studies have shown that PB2 has a positive effect on high glucose-induced mitochondrial dysfunction in cells and improves liver health by promoting lysosomal degradation of substances [13,14,15]. However, its impact on severe inflammatory damage in septic conditions is unknown. This study aimed to examine the mechanism by which PB2 may treat sepsis-induced ALI. In this study, we predicted the underlying mechanism of PB2 in sepsis and established a mouse model of sepsis-induced ALI to test the therapeutic effect of PB2. This research provides insight into the efficacy and mechanism of PB2 against sepsis-induced ALI.

## 2. Results

### 2.1. PB2 Alleviated Acute Lung Injury Induced by Intraperitoneal LPS

**Figure 1:**
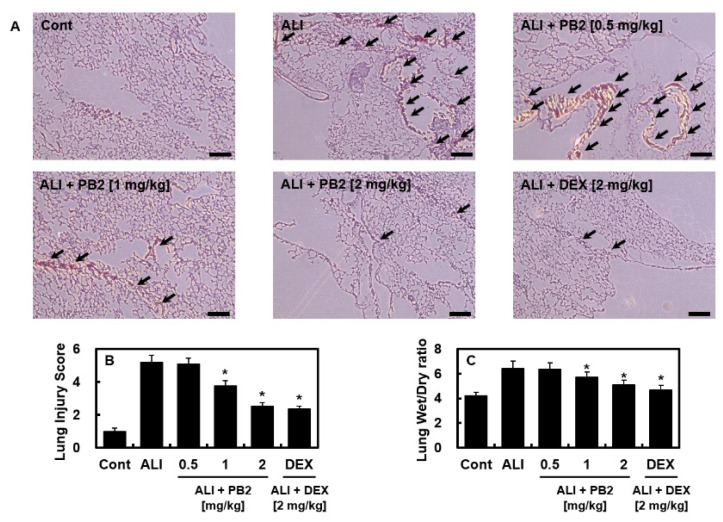
Effects of PB2 on pulmonary pathological changes of sepsis induced ALI mice: (**A**) hematoxylin and eosin (H&E) staining was used to analyze histological changes in the lung tissue. Seven representative photographs from each group were analyzed. Scale bar: 200 μm; (**B**) lung-injury score; and (**C**) lung wet/dry (W/D) weight ratio (*n* = 7/group). The data are shown as mean and SD from three separate studies. * *p* < 0.01 versus ALI group. Arrows indicate pulmonary damages.

First, to determine the optimal concentration of PB2 in the LPS-induced ALI model, concentrations of PB2 ranging from 0.1 mg/kg to 5 mg/kg were used. The results showed that concentrations below 1 mg/kg had no effect, while the effective concentration was above 1 mg/kg, and there was no improvement in efficacy beyond 2 mg/kg. Then, we investigated the effect of PB2 on acute lung injury caused by the LPS-induced sepsis model. At 1 h after LPS injection intraperitoneally, mice were intravenously administrated PB2 or dexamethasone (DEX) twice daily and lung samples were collected 24 h later. The lung damage was evaluated using Hematoxylin and eosin (H&E) staining. The control group had normal and intact structures, while the administration of LPS in the ALI group led to noticeable histological changes, such as congestion, edema, and an influx of inflammatory cells (Figure 1A). However, PB2 treatment significantly alleviated the LPS-induced lung injuries, with the highest dose being the most effective. The ALI group had higher lung-injury scores and lung-weight ratios compared to the PB2-treated groups, with the high-dose group showing the strongest protective effects (Figure 1B,C).

### 2.2. PB2 Reduced the Levels of Inflammatory Cytokines in Blood Serum and Lung Tissues

**Figure 2:**
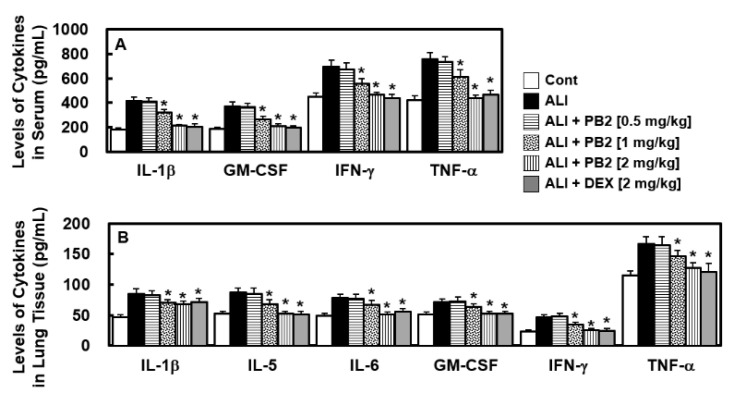
Effects of PB2 on the levels of inflammatory cytokines in sepsis induced ALI mice. Measurements include (**A**) IL-1β, GM-CSF, IFN-γ, and TNF-α in the serum; and (**B**) IL-1β, 1L-5, IL-6, GM-CSF, IFN-γ, and TNF-α in the lung tissues (*n* = 7/group). The data are shown as mean and SD from three separate studies. * *p* < 0.01 vs. ALI group.

Next, we aimed to determine the involvement of inflammatory cytokines, which are crucial in septic responses [16], in the treatment of PB2 on LPS-induced sepsis model. The levels of inflammatory cytokines were measured in the blood serum and lung tissue to determine the effect of PB2. The levels of cytokines, such as IL-1β, GM-CSF, IFN-γ, and TNF-α in the blood serum of the ALI group (Figure 2A) and IL-1β, IL-5, IL-6, GM-CSF, IFN-γ, and TNF-α in the lung tissue of the ALI group (Figure 2B) were found to be significantly higher compared to the control group. However, these levels were found to be lower in the PB2-treated group (Figure 2).

### 2.3. PB2 Inhibited ALI by Regulating the TLR4/NF-κB and PI3K/Akt Signaling Pathways

**Figure 3:**
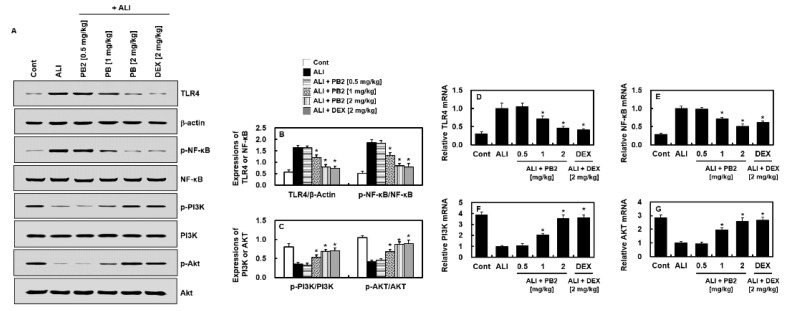
Effects of PB2 on the TLR4/NF-κB and PI3K/Akt signaling pathways in the lung tissue of septic mice; (**A**) typical band images of TLR4, NF-κB, PI3K, and Akt. The prefix “p-” means the phosphorylated form; (**B**,**C**) relative expression of the TLR4/NF-κB and PI3K/Akt pathways; and (**D**–**G**) the mRNA levels of the indicated genes of lungs from the LPS-induced sepsis mice. *n* = 5/group. Data are expressed as the mean ± SD. * *p* < 0.01 vs. ALI group.

Next, we examined the expression of the Toll-like receptor (TLR) signaling pathway, which is known to play a central role in septic responses [17], in lung tissue, using Western blot analysis. The expression levels of TLR4, NF-κB, PI3K, and Akt were compared between the control group and the acute lung-injury (ALI) group. The ALI group showed a significant increase in TLR4 and NF-κB expression and a decrease in PI3K and Akt expression compared to the control group (Figure 3A). In contrast, the PB2-treated groups showed a decrease in TLR4 and NF-κB expression and an increase in PI3K and Akt expression compared to the ALI group (Figure 3B,C), which was confirmed by the mRNA levels (Figure 3D–G). These results suggest that PB2 may prevent the development of ALI by regulating the TLR4/NF-κB and PI3K/Akt signaling pathways.

### 2.4. PB2 Inhibited Hippo and RHOC-ROCK1 Signaling in Sepsis

**Figure 4:**
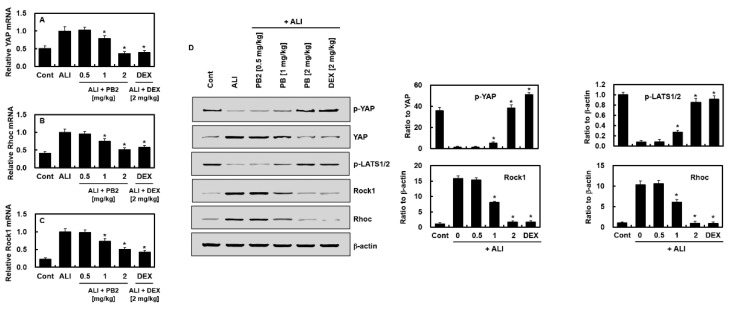
Effects of PB2 on the Hippo -Rho pathway signaling pathways in the lung tissue of septic mice: (**A**–**C**) the mRNA levels of the indicated cytokines in the Hippo pathway and the Rho pathway of lung tissue from the LPS-induced sepsis mice; and (**D**) typical band images of YAP, LATS1/2, ROCK1, and Rhoc (**left**) and densitometric intensities of each signaling-pathway component normalized to β-actin or total protein (**right**). The prefix “p-” means the phosphorylated form. *n* = 5/group. Data are expressed as the mean ± SD. * *p* < 0.01 versus ALI group.

We also investigated whether PB2 could regulate the Hippo signaling pathway to mediate its anti-inflammatory effect. The impact of PB2 on the expression of YAP triggered by LPS was examined, and the findings demonstrated that PB2 could effectively lower the activation of YAP initiated by LPS (Figure 4A). Western blotting was used to evaluate the expression of the Hippo signaling pathway and the Rho family-related proteins in the lung tissues of the LPS-induced sepsis model. An abnormal increase in YAP expression was detected in the lung tissues of septic mice and the levels of YAP and LATS phosphorylation were decreased, which were inhibited by PB2 treatment (Figure 4D). Additionally, the abnormal activation of the Rho family, which is represented by an increase in the mRNA (Figure 4B,C) and protein (Figure 4D) levels of ROCK1 and RHOC, was found in septic lung tissues and inhibited by PB2 treatment.

## 3. Discussion

Sepsis is a dangerous condition due to its complex pathological mechanism. Despite the advancements in medical technologies and anti-infection treatments, there is still a lack of effective drugs for sepsis [18]. Treatment may not always prevent long-term problems in sepsis patients, making it crucial to find effective methods for its prevention, diagnosis, and treatment. The complexity of sepsis calls for natural products with multiple biological activities and targets, as they may offer potential in preventing and treating sepsis. This study found that PB2 has a significant protective effect against sepsis by inhibiting lung injuries, reducing cytokine storm, and regulating the TLR4/NF-κB and PI3K/Akt signaling pathways via its anti-inflammatory properties by inhibiting Hippo and Rho. LPS, a major component of the outer membrane of Gram-negative bacteria, is the most common mediator in the development of sepsis [19]. LPS stimulation causes intense inflammation [20] and small doses can trigger strong immune responses leading to sepsis symptoms [21]. To simulate sepsis-induced lung injury, a high dose of intraperitoneal LPS was used in the study and the lung was chosen as the target organ to examine the effect of PB2 on sepsis.

The results of the animal experiments showed that PB2 significantly decreased the levels of various inflammatory cytokines, such as IL-1β, GM-CSF, IFN-γ, and TNF-α in the blood serum, and IL-1β, IL-5, IL-6, GM-CSF, IFN-γ and TNF-α in the lung tissue of the ALI group. The cause of cytokine storms is still not understood, but it is believed to result from an overactive immune-system activation leading to an imbalance between proinflammatory and anti-inflammatory cytokine production [22]. Excessive cytokine production can drive downstream biological processes, causing fatal respiratory distress and multiple organ failure. The histopathological analysis supported the protective effects of PB2 on the lung tissue from sepsis-induced injury and showed that PB2 reduced pulmonary edema in sepsis, as indicated by the reduced lung W/D weight ratio.

Further research was conducted to understand the mechanism behind PB2’s protective effects against sepsis-induced ALI. The data showed that PB2 decreased the TLR4/NF-κB pathway and increased the PI3K/Akt pathway, as indicated by Western blot analysis. Bacterial LPS can bind and activate TLR4 in the airway epithelium, causing the production of cytokines and other proinflammatory responses [23]. LPS stimulation leads to an increase in TLR4 expression in lung tissue, which activates the NF-κB pathway and amplifies the innate immune response against pathogens. This activation is initiated by the recruitment of MyD88 and/or TRIF to the TLR membrane complex [24,25]. Further investigation into the protective mechanism of PB2 in ALI induced by sepsis was conducted by studying two inflammation-related signaling pathways. Western blot analysis revealed that PB2 decreased the TLR4/NF-κB pathway and increased the PI3K/Akt pathway. The TLR4/NF-κB pathway is activated when the airway epithelium is bound and activated by bacterial LPS, leading to the production of cytokines and other proinflammatory responses [26]. The activation of this pathway has been linked to the onset of ALI. Meanwhile, the PI3K/Akt signaling pathway may act as a compensatory mechanism in response to harmful stimuli and has been shown to reverse pulmonary fibrosis caused by LPS-induced sepsis [27]. However, the exact mechanism by which the PI3K pathway regulates the host response is still not well understood and its pharmacological effect remains controversial. Our study found that the expression of TLR4/NF-κB signaling was increased, and the expression of PI3K/Akt signaling was decreased, in rats with sepsis-induced ALI. PB2 may protect the lung tissue from LPS-induced injury by inhibiting the cytokine storm by downregulating TLR4/NF-κB and upregulating PI3K/Akt.

The PI3K family is categorized into three types, which are Type I, Type II and Type III and these types are involved in fundamental cellular processes such as cell survival, proliferation, differentiation, and metabolism [28,29]. The PI3K/Akt signaling pathway may function as an internal negative-feedback, or compensatory, mechanism related to inflammatory and septic reactions that occur as a result of harmful stimuli [27]. This pathway is closely associated with pulmonary fibrosis, which happens after LPS-induced sepsis in both murine and human cells, and the fibrotic process can be reversed by treatment with the PI3K inhibitor LY294002 [30]. Previous studies suggest that there is a link between TLRs and PI3K regulation of the proinflammatory response. TLRs initiate a proinflammatory signaling cascade and activate transcription-factor families. TLR signals also activate PI3K/Akt through the combination of TLR ligands, which impact many aspects of the cellular response [31]. Although the mechanism behind how the PI3K pathway regulates the host response is still unknown, the pharmacological effect of PI3K remains contentious. While some studies indicate that PI3K contributes to NF-κB activation and promotes the inflammatory response [32], other studies have suggested that PI3K inhibits the inflammatory response. Genetic evidence suggests that the PI3K pathway serves as a crucial negative regulator of the proinflammatory response [33]. The PI3K/Akt/mTOR signaling pathway is a crucial pathway in controlling cell growth and in regulating cellular processes such as proliferation, survival, and metabolism [34,35]. It responds to four primary signals, including energy, nutrients, growth factors, and stress. The pathway is also in competition with the Hippo pathway for dominance [34,35]. LATS1/2 kinases, the primary components of the Hippo pathway, can directly phosphorylate mTORC1, which weakens the activation of the mTORC1 kinase, reducing its activity. The PI3K/Akt/mTOR pathway’s dominance and hyperactivation of NF-κB are linked to different stages of proinflammatory cellular stresses [34,35]. Consequently, these pathways can enter into mutual competition with each other. Understanding the complex interactions between the PI3K/Akt/mTOR and Hippo pathways can provide insight into disease states, such as cancer and inflammation, and the development of targeted therapies. Our research demonstrated that in mice with sepsis-induced ALI, the lung tissue showed an increase in the expression of TLR4/NF-κB signaling and a decrease in the expression of PI3K/Akt signaling. PB2 may protect the lung tissue from LPS-induced injuries by inhibiting the cytokine storm of sepsis through downregulating TLR4/NF-κB and upregulating PI3K/Akt.

Previous research has shown that YAP can worsen inflammation in cases of endo-toxemia and multi-microbial sepsis through the regulation of vascular inflammation and activation of the endothelial cells [36]. Our findings show that the levels of YAP in the lung tissues of mice with sepsis increased, both in mRNA and protein, while the level of phosphorylation decreased. LATS, another key component of the Hippo signaling pathway, showed similar changes, which were reversed by PB2 pretreatment. The Rho family of GTP enzymes plays a role in Hippo signaling activation and is involved in a range of biological processes including cell adhesion, migration, and apoptosis [37]. Based on data showing that PB2 inhibits the activation of the Rho family under ALI conditions, it can be expected that endothelial cytoskeletal rearrangements might be reduced, and fewer neutrophils will be able to migrate into lung tissue. The Hippo signaling pathway is influenced by external stimuli, and the rearrangement of the Rho family is closely tied to YAP. Thus, PB2 may protect the lung tissue by inhibiting the Rho/YAP pathway.

**Figure 5:**
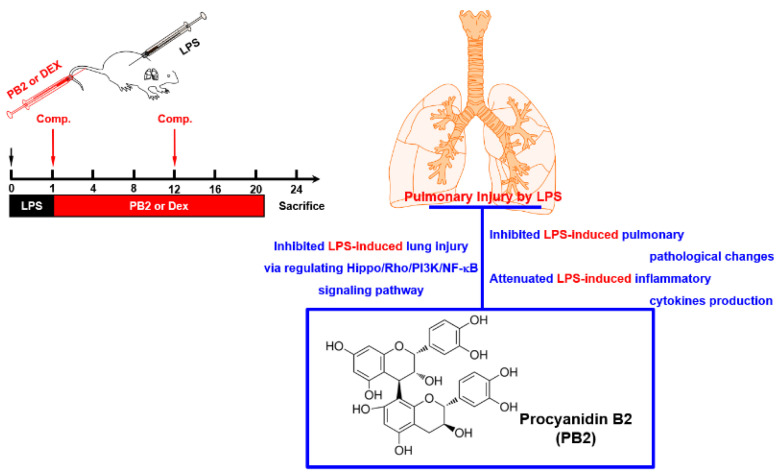
A scheme summarizing the experimental plan and conclusion.

In conclusion, the results from the animal study on mice with LPS-induced ALI showed that PB2 can reduce cytokine storms and mitigate lung damage by controlling the TLR4/NF-κB and PI3K/Akt expression, and by suppressing the activation of the Hippo signaling pathway and the Rho family. PB2 demonstrated anti-inflammatory effects and lung protection against sepsis-induced ALI (Figure 5). This research provides new insights into developing effective treatment strategies for sepsis by targeting cytokine storms and the related signaling pathways.

## 4. Materials and Methods

### 4.1. Materials

PB2 (purity > 90%), DEX (used as a positive control), and LPS derived from bacteria (serotype: 0111:B4, L5293) were purchased from Sigma (St. Louis, MO, USA). Anti-NF-κB, anti-p-NF-κB, anti-Akt, anti-p-Akt, anti-PI3K, anti-p-PI3K, anti-YAP, anti-p-YAP, anti-ROCK, anti-Rhoc, and anti-β-actin antibodies were obtained from Cell Signaling Technology (Danvers, MA, USA). Anti-TLR4 and anti-p-LATS1/2 (phospho-Ser909/872) antibodies were obtained from Santa Cruz Biotechnology (Dallas, TX, USA) and MyBioSource (San Diego, CA, USA), respectively.

### 4.2. Animal Care and Acute Lung Injury Model by LPS Injection

Male C57BL/6 mice with an average weight of 27 g and 6–7 weeks old were obtained from Orient Bio Co. (Sungnam, Republic of Korea) and underwent a 12-day acclimatization period. The mice were kept in polycarbonate cages with five animals per cage and maintained under controlled temperature (20–25 °C) and humidity (40–45%) with a 12-h light and dark cycle. They were given a normal diet and had free access to water. The treatment of all animals followed the Guidelines for the Care and Use of Laboratory Animals published by Kyungpook National University (IRB no. KNU 2022-174). LPS was injected into the mice via intraperitoneal injection at a dose of 5 mg/kg with 0.2% DMSO. One hour and 12 h after the LPS injection, the mice were given intravenous doses of PB2 (0.5, 1, or 2 mg/kg) or DEX (2 mg/kg). The mice in the control group were administered saline via intravenous injection, while both the control and ALI groups of mice were orally administered distilled water by gavage. Lung tissue and blood samples were collected 24 h later.

### 4.3. Hematoxylin and Eosin (H&E) Staining

The lungs of mice were analyzed to observe any changes in their physical appearance. The lungs were extracted from each mouse, washed three times with PBS (pH 7.4) to remove any remaining blood, and then fixed in a 4% formaldehyde solution in PBS, pH 7.4, for 20 h at 4 °C. After fixation, the samples were dehydrated using an ethanol series, embedded in paraffin, and then sectioned into 4-μm sections before being placed on a slide. The slides were then deparaffinized, rehydrated, and stained with hematoxylin (Sigma). To remove any over-staining, the slides were quickly dipped three times in 0.3% acid alcohol, followed by counterstaining with eosin (Sigma). The slides were then washed in an ethanol series and xylene, and finally, cover-slipped. Lung specimens were analyzed using light microscopy, and observations were made by blinded observers to evaluate the pulmonary architecture, tissue edema, and inflammatory cell infiltration as previously defined. The results were classified into four grades, where Grade 1 indicated normal histopathology; Grade 2–3 indicated minimal neutrophil leukocyte infiltration; Grade 4–5 indicated moderate neutrophil leukocyte infiltration, perivascular edema formation, and partial destruction of pulmonary architecture; and Grade 6 indicated dense neutrophil leukocyte infiltration, abscess formation, and complete destruction of pulmonary architecture, following a standard protocol as described in [38].

### 4.4. Lung Wet/Dry (W/D) Weight Ratios

The lung wet-to-dry (W/D) weight ratio was utilized as a way to determine the amount of water accumulation in the lungs after ATP was administered. To measure the total quantity of lung water, the animals were dissected while under deep sevoflurane anesthesia and the weight of the lung was measured immediately after removal (wet weight). The lung tissue was then dried in an oven at 120 °C for 1 day and re-weighed as dry weight. The W/D weight ratio was determined by dividing the wet weight by the dry weight.

### 4.5. Detection of Inflammatory Cytokines

Samples of whole blood from the abdominal aorta and the middle lobe of the right lung were collected to measure levels of inflammatory cytokines. The blood was centrifuged at 1500× *g* for 10 min, and the serum in the upper layer was collected. The tissue was lysed with a buffer and protease inhibitors (Sigma), crushed with ultrasonic waves, and then centrifuged at 13,000× *g* for 15 min (4 °C). The BCA protein assay kit (Pierce, CA, USA) was used to measure the protein concentration in each tissue sample. The concentrations of various inflammatory cytokines, such as interleukin (IL)-1β, IL-5, IL-6, granulocyte-macrophage colony-stimulating factor (GM-CSF), interferon (IFN)-γ, and tumor necrosis factor (TNF)-α, were measured in the blood serum and lung tissue using commercially available ELISA kits, according to the manufacturer’s instructions (R&D Systems, Minneapolis, MN, USA).

### 4.6. Western Blot

The right lung tissue was extracted and then subjected to a lysis process using a Radioimmunoprecipitation assay (RIPA, containing 150 mM NaCl, 1.0% IGEPAL^®^ CA-630, 0.5% sodium deoxycholate, 0.1% SDS, 50 mM Tris, pH 8.0., purchased from Sigma) buffer and protease inhibitor contail (AEBSF at 2 mM, Aprotinin at 0.3 μM, Bestatin at 116 μM, E-64 at 14 μM, Leupeptin at 1 μM and EDTA at 1 mM, purchased from Sigma). The lysate was centrifuged at 13,000× *g* for 15 min at 4 °C. The protein content was measured using a BCA protein assay kit (Pierce, CA, USA). A sample of 40 μg of protein was separated using 10% sodium dodecyl sulfate-polyacrylamide gel electrophoresis, transferred onto PVDF membranes (Millipore, Darmstadt, Germany), and incubated with diluted primary antibodies overnight at 4 °C. Afterward, the secondary antibody was added, and the target proteins were visualized using enhanced chemiluminescence (Millipore, MA, USA). β-actin was used as an internal control. The chemiluminescence imaging system was utilized to visualize the immune complexes. The ImageJ Gel Analysis tool (NIH, Bethesda, MD, USA) was used for concentration analyses.

### 4.7. Total RNA Isolation and Quantitative Real-Time PCR (qPCR)

Frozen lung-tissue samples were placed in 2 mL ceramic bead tubes (VWR, Randor, PA, USA) containing 500 µL of Qiazol lysis reagent. The tubes were homogenized for 120 s at 4 m/s using a Mini Bead Mill Homogenizer, and then centrifuged at 12,000 rcf at 4 °C for 15 min. The supernatants were collected, and total RNA was extracted using the RNeasy mini and DNase I kits (Qiagen, Germantown, MD, USA). The purity and concentration of total mRNA were determined using a Nanodrop (ThermoFisher Scientific, Waltham, MA, USA), and RNA preparations with an A260/A280 ratio between 1.8 and 2.2 were considered acceptable. First-strand cDNA was generated from 600 ng of total RNA using the iScript Advanced cDNA synthesis kit (Bio-Rad, Hercules, CA, USA) according to the manufacturer’s instructions. qPCR amplification was conducted on a CFX96 Real-Time System made by Bio-Rad Laboratories (Hercules, CA, USA). CFX Manager ver. 1.6 software (Bio-Rad) was used to track fluorescence signals. The software was also used for data analysis. The qPCR assay was performed in a 20 µL optimized reaction system, including 10 µL of 2× Premix Ex Taq (probe qPCR, Takara, Kusatsu, Shiga, Japan) 0.4 µM primers, 0.2 µM probe, and 100 ng of genomic DNA or 5.2 × 10^3^ copies of plasmid DNA as a template. The TaqMan probes used in these qPCR experiments were labelled with the fluorescent reporter 6-carboxy-fluorescein (FAM) and the non-fluorescent Black Hole Quencher 1 (BHQ1). The duplex qPCR assay had a reaction mixture (20 µL) that contained 2× Premix Ex Taq (probe qPCR) (Takara, Kusatsu, Shiga, Japan), 0.4 µM of qHptF286/qHptR395 and Ghpt-QF3/Ghpt-QR3, 0.2 µM of HptR697 and Ghpt-QP, and 50 ng of genomic DNA or plasmid DNA (500 copies) as a template. All reactions were tested in triplicate with the same program, which included an initial denaturation step for 1 min at 95 °C, followed by 40 cycles or 50 cycles of 5 sec at 95 °C (denaturation) and 30 sec at 60 °C (annealing and extension). The fluorescence signal was measured after each annealing and extension step. The primer sequences used in the reaction are listed in Table 1. The gene expression of each gene was compared to β-actin, which served as a reference.

### 4.8. Statistical Analysis

The results were presented as the average and standard deviation (SD) of at least three experiments. SPSS software version 16.0 (SPSS, Chicago, IL, USA) was used to compare the differences between groups. One-way ANOVA, and Tukey’s post hoc test, were performed to determine statistical significance. A significance level of *p* < 0.05 was considered.

## Figures and Tables

**Table 1:** Specific primer sequences used for quantitative real-time PCR.

Gene	Primer Sequences
*β-actin* (F)	ACCGTGAAAAGATGACCCAG
*β-actin* (R)	GTACGACCAGAGGCATACAG
*YAP* (F)	CAGGTATTGGGAGAGTCACGG
*YAP* (R)	CAAGGGGATGACTCCAGTGAG
*RHOC* (F)	CCATGGCTGCGATCCGAA
*RHOC* (R)	GGTAGGCACGTAGACCTCTG
*Rock-1* (F)	AAGCCGCACTGATGGATATGT
*Rock-1* (R)	GCCATCTATTCATTCCAGCCAT
*TLR4* (F)	TTCATGTCGTGTTCTCATGG
*TLR4* (R)	TGCGCTCGCATCATGTTC
*NF-κB* (F)	GCAAAGGGAACATTCCGATAT
*NF-κB* (R)	GCGACATCACATGGAAATCTA
*PI3K* (F)	GTGTCAGCGCTCTCCGCC
*PI3K* (R)	AGCGACCCTGTACCAAGTT
*AKT* (F)	GTGTCCAGTGTAGAATGACTC
*AKT* (R)	ATCTGTCGGAGAACACACATG

## Data Availability

The data presented in this study are available upon reasonable request from the corresponding author.
